# Surgical Procedure of Lateral Lymph Node Dissection for Advanced Lower Rectal Cancer

**DOI:** 10.1002/ags3.70039

**Published:** 2025-05-13

**Authors:** Mamoru Uemura, Jun Watanabe, Akio Shiomi, Takashi Akiyoshi, George J. Chang, Yukihide Kanemitsu, Ichiro Takemasa, Yoshiharu Sakai, Masahiko Watanabe, Itaru Endo

**Affiliations:** ^1^ Department of Gastroenterological Surgery Graduate School of Medicine, The University of Osaka Osaka Japan; ^2^ Department of Surgery Gastroenterological Center Yokohama City University Medical Center Yokohama Japan; ^3^ Department of Colorectal Surgery Kansai Medical University Osaka Japan; ^4^ Division of Colon and Rectal Surgery Shizuoka Cancer Center Hospital Shizuoka Japan; ^5^ Department of Colorectal Surgery, Gastroenterological Center Cancer Institute Hospital, Japanese Foundation for Cancer Research Tokyo Japan; ^6^ Department of Colon and Rectal Surgery University of Texas MD Anderson Cancer Center Houston Texas USA; ^7^ Department of Colorectal Surgery National Cancer Center Hospital Tokyo Japan; ^8^ Department of Gastroenterological Surgery Osaka International Medical and Science Center, Osaka Keisatsu Hospital Osaka Japan; ^9^ Department of Surgery Osaka Red‐Cross Hospital Osaka Japan; ^10^ Department of Surgery Kitasato University Sagamihara‐shi Kanagawa Japan; ^11^ Department of Gastroenterological Surgery Yokohama City University Yokohama Kanagawa Japan

**Keywords:** lateral lymph node dissection, lower rectal cancer, surgical procedure

## Abstract

Lateral lymph node dissection (LLND) is recognized as an effective treatment for reducing local recurrence in patients with locally advanced lower rectal cancer. However, the lack of standardization in techniques and anatomical landmarks remains a concern, as it may complicate the assessment of treatment efficacy. To address this, the Japan Society of Gastroenterological Surgery (JSGS) held a consensus meeting during the 77th General Meeting of the JSGS to standardize LLND techniques. In this meeting, essential anatomical landmarks for LLND were confirmed. The primary regions targeted for dissection include lymph nodes in the obturator region (designated as station 283) and those in the internal iliac region (designated as station 263). The medial boundary of LLND is defined by the uretero‐hypogastric fascia, whereas the vesico‐hypogastric fascia constitutes the central plane of dissection and serves as the medial boundary of station 283. Indicators of successful LLND completion include exposure of the sciatic nerve (lumbosacral trunk) at the bottom of the dissection, as well as exposure of the inferior vesical vessels, internal pudendal artery, and coccygeus muscle, confirming the thoroughness of the caudal part of the dissection. The consensus reached in this meeting, along with findings from several published reports cited in this report, is expected to contribute to the standardization of LLND quality.

## Graphical abstract

Lateral lymph node dissection (LLND) is an effective surgical approach for lower rectal cancer. This paper outlines key points from the Japan Society of Gastroenterological Surgery consensus meeting, including standardized LLND techniques and procedural highlights.

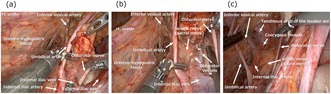

## 1 Introduction

In Western countries, neoadjuvant chemoradiotherapy or radiotherapy ((C)RT) [1, 2, 3] or total neoadjuvant therapy (TNT) combined with total mesorectal excision [4] is the standard treatment for locally advanced lower rectal cancer. Historically, the lateral pelvic lymph nodes have been overlooked, and lateral lymph node dissection (LLND) has been omitted in Western countries. Even in recent years, LLND is only performed in a limited number of specialized centers when there are overtly suspicious lymph nodes [5]. However, in cases where enlarged lateral pelvic lymph nodes are detected after preoperative (C)RT, LLND remains an important option for reducing the risk of local recurrence [6, 7].

In contrast, in Japan, LLND has traditionally been performed without neoadjuvant treatment for locally advanced lower rectal cancer. The Japan Clinical Oncology Group 0212 (JCOG0212) study compared mesenteric excision (ME) alone with ME plus LLND and found that the frequency of local recurrence was significantly reduced in the ME plus LLND group, suggesting an oncological benefit of LLND for reducing local recurrence in clinical stage II/III lower rectal cancer [8].

Numerous studies have demonstrated that LLND is a crucial component in the treatment of locally advanced lower rectal cancer. High‐quality LLND is essential not only for achieving local control but also for minimizing nerve injury and managing intraoperative bleeding. Despite its importance, mastering this technique remains a significant challenge because of the lack of standardized procedures and consistent definitions regarding the extent of lymph node dissection [9]. Moreover, this variability complicates comparisons and evaluations of treatment outcomes.

In this context, the Japan Society of Gastroenterological Surgery (JSGS) held a consensus meeting to establish standardized procedures for LLND, with the goal of improving treatment consistency and the reliability of outcomes. In this paper, we present the standardized LLND procedures discussed at this meeting and review the current status of LLND as reported in recent studies.

## 2 Consensus Meeting on the Surgical Procedure of LLND at JSGS 2022

At the 77th General Meeting of the Japanese Society of Gastroenterological Surgery (JSGS), a consensus meeting was held to standardize the surgical procedure of LLND for advanced lower rectal cancer. This meeting, titled “Consensus Meeting 2: Surgical Procedure of Lateral Lymph Node Dissection,” included video demonstrations of both laparoscopic and robotic LLND techniques. In the subsequent discussion, the anatomical landmarks and the extent of dissection necessary for a common understanding were confirmed. The following sections outlined the main discussion points, agreements reached, and the standardized approach for laparoscopic and robotic LLND.

## 3 Basic Anatomical Landmarks

Prior to discussing the procedure for LLND, the basic anatomy and anatomical landmarks were reviewed. The medial border of LLND is defined by the uretero‐hypogastric fascia [10], whereas the lateral border is formed by the pelvic wall, consisting of the psoas and internal obturator muscles. Another key landmark is the vesico‐hypogastric fascia [10], located centrally within the dissection area. This intermediate plane contains the internal iliac vessels that supply the pelvic organs. The bottom of the dissection area is formed by the sciatic (lumbosacral trunk) nerve and the coccygeus muscle. At the most caudal end of the dissection, the tendinous arch of the levator ani and the levator ani muscle serve as crucial landmarks.

Key vascular landmarks include the internal and external iliac vessels, the umbilical artery, the superior vesical vessels, and the inferior vesical vessels. The obturator vessels are generally divided, whereas the obturator nerve is preserved. In cases where lymphadenectomy for metastatic nodes is challenging due to vessel preservation or fibrosis from preoperative treatment, the branches of the internal iliac vessels may be resected together with the lateral lymph nodes.

## 4 Extent of LLND

According to the Japanese Society for Cancer of the Colon and Rectum, the lymph nodes in the obturator region are designated as station 283, and those in the internal iliac region as station 263 [11]. Dissection of these two lymph node stations is fundamental to the LLND procedure. Station 283, which corresponds to the obturator region, has its cranial border defined by the external iliac vein and the psoas muscle, its lateral border by the internal obturator muscle, and its medial border by the vesico‐hypogastric fascia.

Station 263 corresponds to the internal iliac area, with the medial border defined by the ureter and the uretero‐hypogastric fascia. Station 263 is subdivided into a distal part (263D) and a proximal part (263P). Although there is no precise definition for the boundary between 263D and 263P, the superior vesical artery typically serves as the dividing line. The cranial end of 263P is generally considered to be the bifurcation of the internal iliac artery.

Due to the relatively high frequency of lymph node metastasis in station 263D, thorough lymphadenectomy in this region is crucial [10]. Landmarks indicating a complete dissection include the internal pudendal artery and the inferior vesical artery, which run dorsally to the coccygeus muscle. It is essential that these landmarks are fully exposed, and the surrounding fat tissue, including lymph nodes, is adequately removed.

The decision to resect vessels, particularly the inferior vesical vessels, during LLND depends on whether their removal is necessary to achieve adequate lymphadenectomy for metastatic nodes and whether safe preservation is feasible, considering the effects of inflammation or tissue fibrosis induced by preoperative treatment. Vessel preservation is generally preferred when feasible.

Dissection in the common iliac area, designated as station 273, is performed by some institutions based on the concept of ensuring thorough clearance of target lymph node regions by starting from the outer area. However, the reported metastasis rate in station 273 is only 0.4% [12], suggesting that it is not an area routinely requiring dissection. Considering the frequency of metastasis, the clinically significant stations for dissection should include at least 263 and 283.

## 5 Laparoscopic and Robotic Surgical Procedures for LLND

The step‐by‐step procedure of prophylactic nerve‐preserving LLND, as presented during the consensus meeting, is described using intraoperative pictures of right‐sided LLND.

**FIGURE 1:**
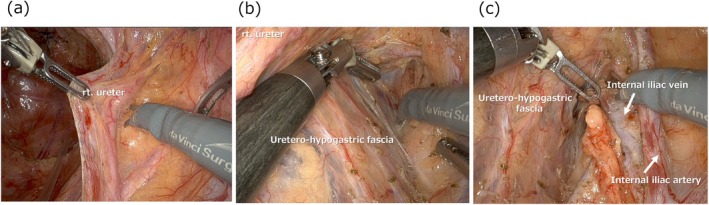
Ureter dissection and dissection along the uretero‐hypogastric fascia, followed by lymphadenectomy in the internal iliac region.

In LLND, the initial step is to carefully dissect the ureter away from the surrounding tissues to preserve its integrity. The ureter is gently retracted medially, with careful attention to avoid thermal injury and to preserve the small blood vessels surrounding it (Figure 1a). With adequate traction, the boundary between the fat tissue targeted for dissection and the uretero‐hypogastric fascia becomes clearly defined (Figure 1b). During this process, it is essential to proceed cautiously to avoid damaging the pelvic plexus that comprises the uretero‐hypogastric fascia. For the dissection of lymph nodes in the internal iliac region, the dorsal line of the internal iliac vein serves as an anatomical guide (Figure 1c).

**FIGURE 2:**
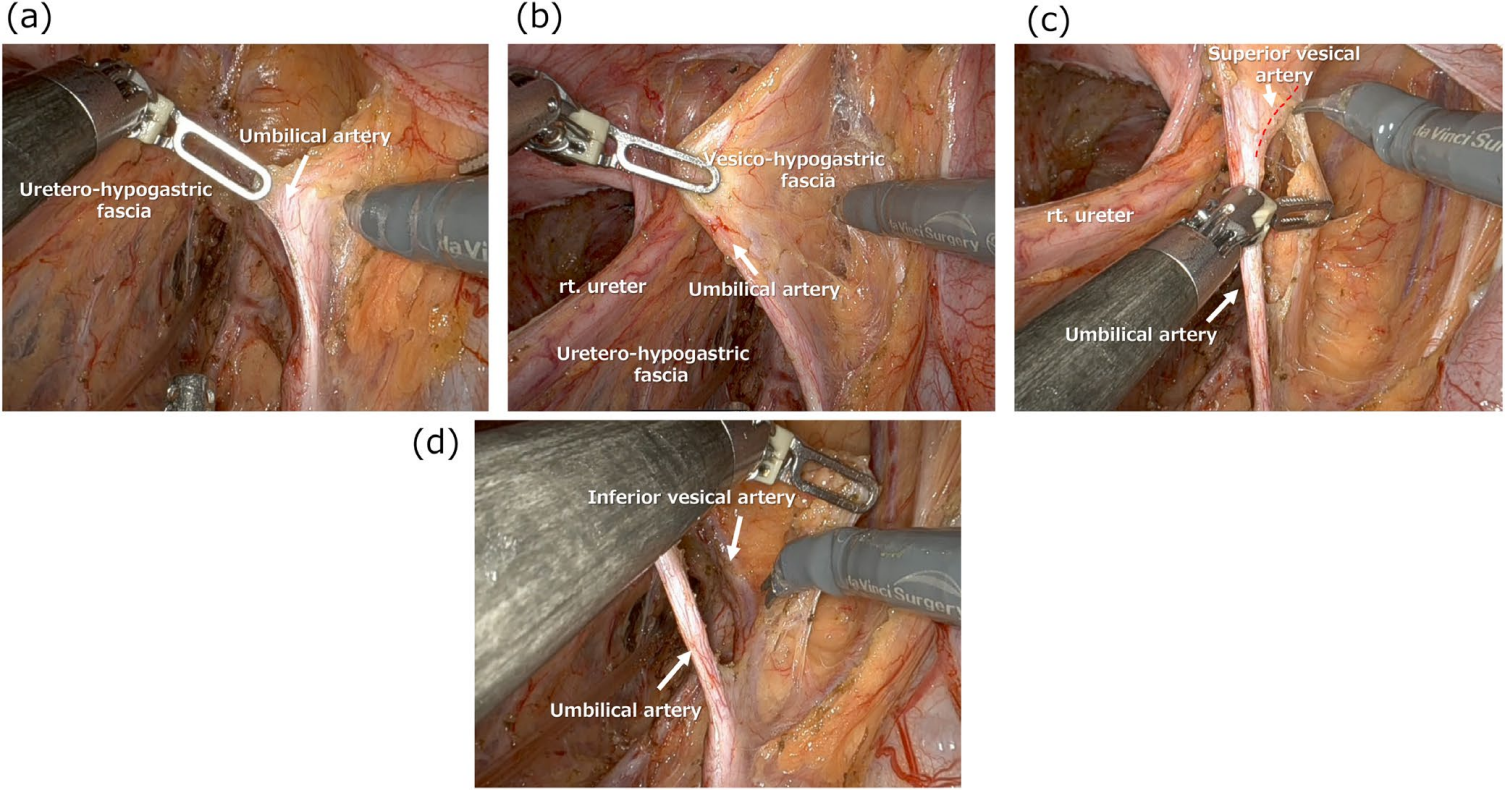
Clarification of the obturator region via dissection along the vesico‐hypogastric fascia and lymphadenectomy for the distal part of the internal iliac region.

The medial boundary of the obturator region is defined by the vesico‐hypogastric fascia. By retracting the umbilical artery medially and applying steady traction (Figure 2a), the boundary between the fat tissue to be dissected and the vesico‐hypogastric fascia becomes clearly visible, enabling precise dissection along this plane (Figure 2b). To ensure thorough lymph node removal in the internal iliac region, an incision is made in the vesico‐hypogastric fascia to create a window, exposing the superior vesical artery at the top and the internal iliac vessels at the bottom (Figure 2c). During this procedure, it is important to achieve clear exposure of the inferior vesical vessels located in the deeper (caudal) part of the dissection space (Figure 2d), allowing precise separation between the fatty tissue to be dissected and the vessels that must be preserved.

**FIGURE 3:**
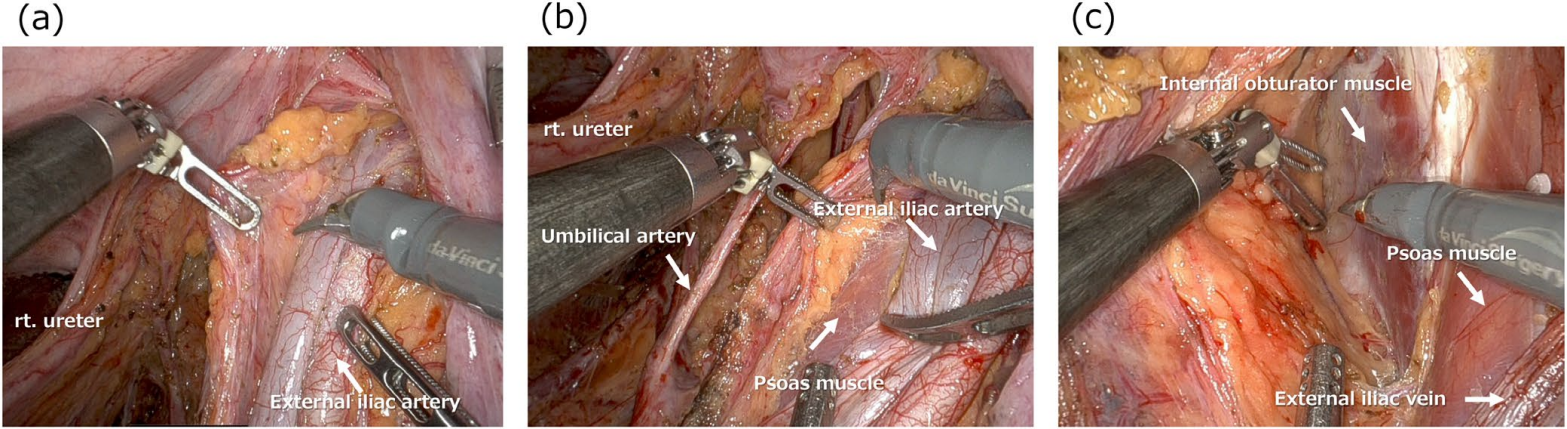
Exposure of the cranial and lateral borders in LLND.

The cranial boundary of the LLND is typically defined by the external iliac vessels. To ensure complete dissection of the obturator region, the external iliac artery is first exposed, followed by exposure of the external iliac vein running medially to it (Figure 3a). Distal landmarks for the dissection level of the external iliac vessels include the branching points of the accessory internal obturator vein and the inferior epigastric vessels. By retracting the external iliac vein cranially and carefully dissecting along the medial edge of the external iliac artery, the psoas muscle is easily reached (Figure 3b). As dissection continues caudally along the surface of the psoas muscle, the surface of the internal obturator muscle is gradually exposed (Figure 3c). The surface of the internal obturator muscle, which forms the lateral boundary of the LLND, is then widely exposed.

**FIGURE 4:**
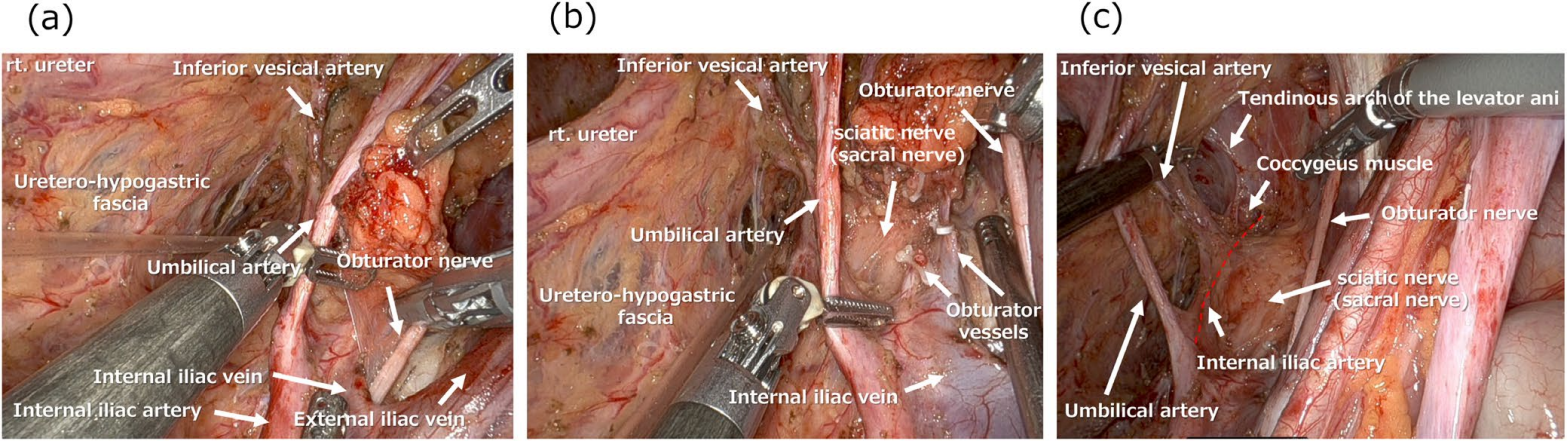
Procedures at the bottom of LLND and landmarks indicating completion of lymphadenectomy at the deepest point.

The obturator nerve, which is typically preserved, runs between the bifurcation of the internal and external iliac veins. As this bifurcation is exposed, the obturator nerve can be identified posterior to it (Figure 4a). The nerve is carefully dissected free from surrounding fat tissue and retracted laterally to create the bottom of the dissection plane. The obturator vessels are typically divided (Figure 4b). The surface of the sciatic nerve (sacral nerve) serves as a landmark for the bottom of the dissection, and dissection proceeds caudally along its surface (Figure 4b). At the most distal part of the dissection area, the internal pudendal artery, a distal branch of the internal iliac artery, is identified. The internal pudendal artery runs just dorsal to the coccygeus muscle and passes through the infrapiriform foramen. Clear exposure of the distal part of the internal iliac artery (internal pudendal artery) and the coccygeus muscle serves as a key landmark indicating complete dissection in this region (Figure 4c). In the deepest part of the dissection field, the inferior vesical vessels are identified medially, whereas laterally, the tendinous arch of the levator ani and the levator ani muscle behind it serve as indicators of a completed dissection (Figure 4c).

In laparoscopic or robotic LLND, the dissection of the bottom part is completed before proceeding to the final ventral dissection. If the ventral dissection is attempted earlier, tissue sagging toward the posterior can make further dissection difficult. Anatomical landmarks for the ventral side of LLND include the accessory obturator vein, the pubic bone, and the spermatic duct or round ligament of the uterus.

## 6 Additional Topics Related to LLND

For LLND in rectal cancer, various approaches are available depending on the surgeon's familiarity and preference, including open surgery, laparoscopic, robotic, and transanal endoscopic approaches in combination with laparoscopic or robotic techniques. At present, no single approach is definitively recommended. Similarly, a consensus has not been reached regarding the optimal energy devices for this procedure.

Additionally, several studies have reported on the use of indocyanine green fluorescence imaging to visualize lymphatic flow and lymph nodes. However, the current evidence remains insufficient to support its utility in identifying nodes for dissection or distinguishing metastatic nodes in LLND. It remains essential to ensure complete resection of the targeted lymph nodes along with surrounding adipose tissue in the designated regions.

## 7 Current Status of LLND in Recent Literature

### 7.1 Indication of Prophylactic LLND

In Japan, preoperative chemoradiotherapy (C)RT has not been established as a standard treatment for locally advanced lower rectal cancer, and LLND has been positioned as the standard surgical approach. The significance of LLND in cases without enlarged lymph nodes was examined in the JCOG0212 study, a randomized controlled trial conducted in Japan, involving 701 patients. The JCOG0212 study aimed to confirm the non‐inferiority of mesorectal excision (ME) alone compared to ME with LLND in patients with stage II/III rectal tumors located below the peritoneal reflection and without enlarged lateral lymph nodes (< 10 mm in short‐axis). The results did not confirm the non‐inferiority of ME alone; ME with LLND demonstrated lower rates of local recurrence compared to ME alone (7% vs. 13%) [8]. Based on these findings, LLND has retained its position as the standard treatment for locally advanced lower rectal cancer in Japan.

Based on this background, the guidelines for colorectal cancer treatment by the Japanese Society for Cancer of the Colon and Rectum [13] recommend LLND for rectal cancer cases where the tumor's lower margin is located distal to the peritoneal reflection and has invaded beyond the muscularis propria. A retrospective multicenter study in Japan reported that the incidence of lateral pelvic lymph node metastasis in patients with T3 or T4 lower rectal cancer was 18.1% [14]. Furthermore, it was reported that even in cases without lymph nodes enlarged to 10 mm or more in short‐axis diameter, the probability of metastasis in rectal cancers meeting these criteria was 7.4% [8], suggesting that LLND is effective for local control.

The findings of the JCOG0212 study cannot be applied to cases that received preoperative treatment. This is because the eligibility criteria for JCOG0212 required that neoadjuvant treatment, which is standard in Western countries, not be administered. In Western countries, preoperative (C)RT has been shown to reduce the risk of local recurrence [2], and thus LLND is not considered a standard treatment for locally advanced lower rectal cancer. The European Society for Medical Oncology guidelines state that (C)RT is superior to LLND. However, it is mentioned that this recommendation is not based on results from high‐quality trials [15]. There is a belief that some lateral lymph node metastases may regress with preoperative (C)RT, and the American Society of Colon and Rectal Surgeons strongly recommends against performing LLND in cases without enlarged lymph nodes [16]. In cases without enlarged lymph nodes, prophylactic LLND may result in overtreatment if preoperative (C)RT is performed; however, established criteria for decision‐making are currently lacking.

### 7.2 Variations of LLND

The decision on whether to perform unilateral or bilateral LLND is sometimes debated, particularly in cases where preoperative diagnosis suggests unilateral lateral lymph node metastasis. Performing LLND on only one side facilitates the preservation of autonomic nerves on the contralateral side, making selective unilateral LLND a viable option in certain cases [17].

A study comparing the outcomes of unilateral and bilateral LLND for advanced lower rectal cancer reported that, based on multivariate analysis, the relative risk of local recurrence was four times lower in patients who underwent bilateral LLND compared to those who had unilateral LLND [17]. However, as this study was conducted at a single institution with a sample size of approximately 200 cases, it is difficult to draw definitive conclusions.

**FIGURE 5:**
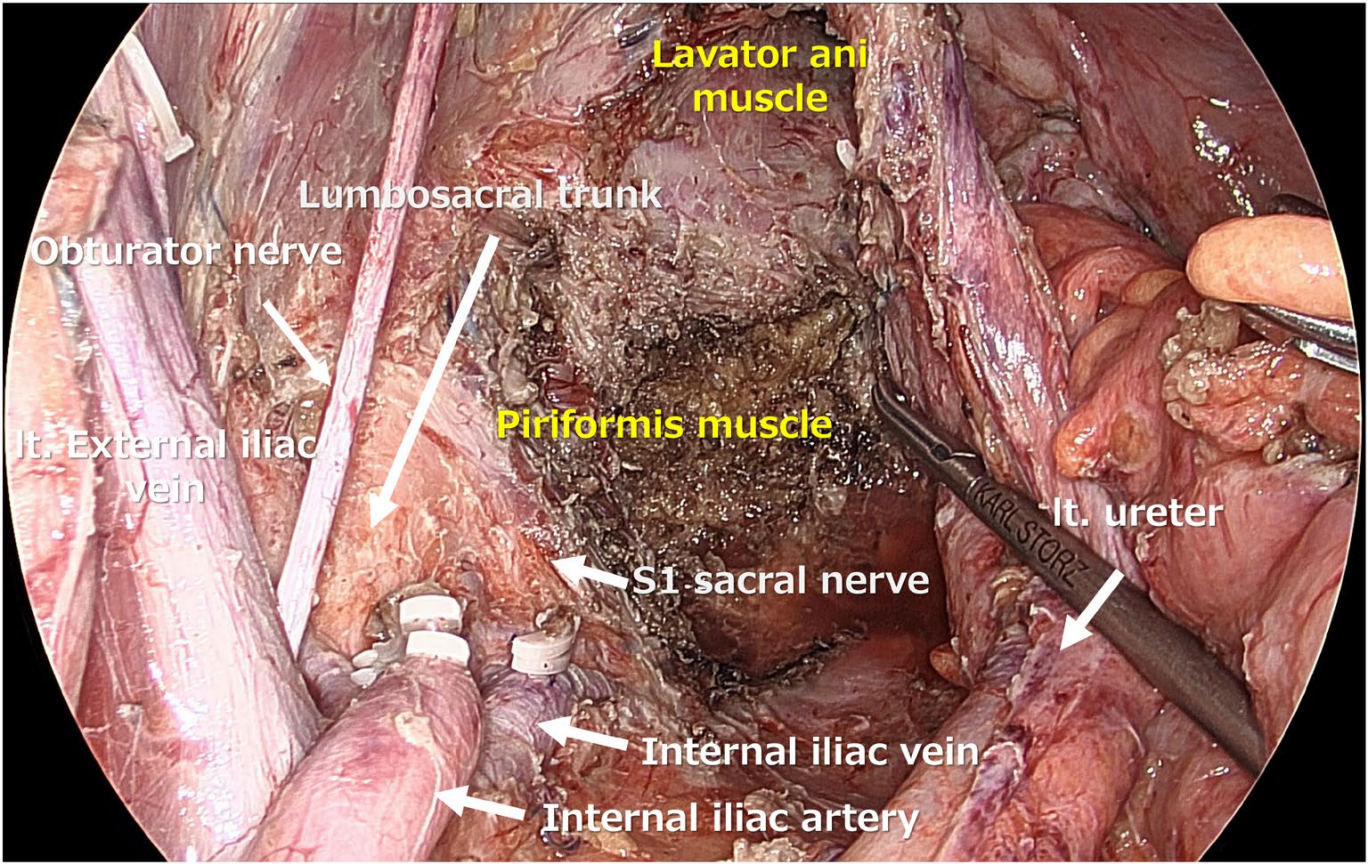
Surgical view after laparoscopic left extended LLND with en bloc resection of the internal iliac vessels, sacral nerves, and piriformis muscle for a case of lateral lymph node recurrence.

In cases with evident lateral lymph node metastasis, oncological safety should be the primary consideration, and vascular and nerve‐sacrificing procedures, such as resection of the internal iliac vessels and obturator nerve, should not be avoided when necessary. Particularly in cases of lateral lymph node recurrence, extended LLND with resection of the internal iliac vessels is usually required. The safety and efficacy of laparoscopic extended LLND have also been reported [18]. In cases where extended LLND with vascular resection is performed, the absence of the internal iliac vessels after dissection allows for clear identification of the sacral nerves dorsally. Furthermore, if the sacral nerves are resected, the piriformis muscle can be observed posterior to them (Figure 5).

### 7.3 Efforts to Establish a Common Understanding Across Multiple Disciplines in Performing LLND

Cross‐departmental efforts have been undertaken to standardize LLND by unifying anatomical landmarks, terminology, and dissection extents. This initiative, involving the departments of gastroenterological surgery, urology, and gynecology, aims to establish a consistent approach to LLND. Although each department has specific indications for the procedure, there are shared and department‐specific aspects in the technique and extent of dissection. In urology and gynecology, LLND is more commonly referred to as “pelvic lymph node dissection.” Through this collaborative project, the obturator region (referred to as station 283 in gastroenterological surgery) has been designated as a standard common dissection area, with the vesico‐hypogastric fascia as the medial boundary and the sciatic nerve (sacral nerve) and coccygeus muscle forming the floor of the dissection [10].

## 8 Discussion

Historically, the treatment strategies for rectal cancer in Eastern and Western countries have been distinctly different, with LLND being performed in Eastern countries and not typically in Western countries. However, there is an emerging shift toward a more individualized approach, where patient selection is becoming increasingly important in deciding the indication for LLND [19].

In Japan, where up‐front surgery with LLND has traditionally been the standard treatment for advanced lower rectal cancer, preoperative therapy is increasingly being adopted for cases with a high risk of recurrence [13]. Moreover, many institutions have started conducting clinical trials to evaluate TNT [20, 21].

Since preoperative therapy is expected to eradicate some degree of LLN metastasis, the opportunity to omit LLND is increasing even in Eastern countries. However, in cases of local recurrence, especially in lateral lymph node metastatic recurrence, LLND remains essential. In such cases, previous surgeries and prior (C)RT treatments often lead to adhesion, fibrosis, and tissue sclerosis, increasing the complexity of the procedure [22]. Performing LLND in these challenging cases requires mastery of basic LLND techniques, along with a standardized recognition of anatomical landmarks and a consistent understanding of the dissection range. On the other hand, adhering strictly to standardized procedures may compromise oncological outcomes in cases of lateral lymph node recurrence or those requiring vascular resection. Therefore, the surgical approach should be adjusted based on the extent and pattern of lateral lymph node metastasis in each case.

The consensus reached in this meeting, along with findings from several reports cited in this report, is expected to contribute to the standardization of LLND quality.

## Author Contributions

**Mamoru Uemura:** supervision, writing – review and editing. **Jun Watanabe:** conceptualization, writing – review and editing. **Akio Shiomi:** conceptualization, writing – review and editing. **Takashi Akiyoshi:** conceptualization, writing – review and editing. **George J. Chang:** supervision, writing – review and editing. **Yukihide Kanemitsu:** conceptualization, writing – review and editing. **Ichiro Takemasa:** conceptualization, writing – review and editing. **Yoshiharu Sakai:** conceptualization, writing – review and editing. **Masahiko Watanabe:** conceptualization, supervision, writing – review and editing. **Itaru Endo:** conceptualization, supervision, writing – review and editing.

## Conflicts of Interest

I.E., J.W., and G.J.C. are members of the Annals of Gastroenterological Surgery Editorial Board.
